# Social mobilisation, consent and acceptability: a review of human papillomavirus vaccination procedures in low and middle-income countries

**DOI:** 10.1186/s12889-016-3517-8

**Published:** 2016-08-19

**Authors:** Severin Kabakama, Katherine E. Gallagher, Natasha Howard, Sandra Mounier-Jack, Helen E. D. Burchett, Ulla K. Griffiths, Marta Feletto, D. Scott LaMontagne, Deborah Watson-Jones

**Affiliations:** 1 Mwanza Intervention Trials Unit, National Institute for Medical Research, PO Box 11936, Mwanza, Tanzania; 2 Clinical Research Department, London School of Hygiene and Tropical Medicine, Keppel St, London, WC1E 7HT UK; 3 Department of Global Health and Development, London School of Hygiene and Tropical Medicine, Tavistock Place, London, WC1H 9SH UK; 4 PATH, Vaccine Access and Delivery, PO Box 900922, Seattle, WA 98109 USA

**Keywords:** HPV, Vaccination, LMICs, Acceptability, Mobilisation, Communication, Consent

## Abstract

**Background:**

Social mobilisation during new vaccine introductions encourages acceptance, uptake and adherence to multi-dose schedules. Effective communication is considered especially important for human papillomavirus (HPV) vaccine, which targets girls of an often-novel age group. This study synthesised experiences and lessons learnt around social mobilisation, consent, and acceptability during 55 HPV vaccine demonstration projects and 8 national programmes in 37 low and middle-income countries (LMICs) between January 2007 and January 2015.

**Methods:**

A qualitative study design included: (i) a systematic review, in which 1,301 abstracts from five databases were screened and 41 publications included; (ii) soliciting 124 unpublished documents from governments and partner institutions; and (iii) conducting 27 key informant interviews. Data were extracted and analysed thematically. Additionally, first-dose coverage rates were categorised as above 90 %, 90–70 %, and below 70 %, and cross-tabulated with mobilisation timing, message content, materials and methods of delivery, and consent procedures.

**Results:**

All but one delivery experience achieved over 70 % first-dose coverage; 60 % achieved over 90 %. Key informants emphasized the benefits of starting social mobilisation early and actively addressing rumours as they emerged. Interactive communication with parents appeared to achieve higher first-dose coverage than non-interactive messaging. Written parental consent (i.e., opt-in), though frequently used, resulted in lower reported coverage than implied consent (i.e., opt-out). Protection against cervical cancer was the primary reason for vaccine acceptability, whereas fear of adverse effects, exposure to rumours, lack of project/programme awareness, and schoolgirl absenteeism were major reasons for non-vaccination.

**Conclusions:**

Despite some challenges in obtaining parental consent and addressing rumours, experiences indicated effective social mobilisation and high HPV vaccine acceptability in LMICs. Social mobilisation, consent, and acceptability lessons were consistent across world regions and HPV vaccination projects/programmes. These can be used to guide HPV vaccination communication strategies without additional formative research.

## Background

Low and middle-income countries (LMICs) carry the highest burden of cervical cancer, accounting for 85 % of global incidence and 90 % of related mortality [1, 2]. Gardasil® and Cervarix® became available in 2006–2007 as vaccines against human papillomavirus (HPV), the main causative agent of cervical cancer [3, 4]. HPV vaccination has become a key component of comprehensive cervical cancer prevention and by August 2015 was being delivered in over 80 countries and territories worldwide in ‘demonstration projects’ or national programmes [1, 4–6].

To introduce a new vaccine successfully, early and active engagement of policy-makers, practitioners, and communities is necessary. Social mobilisation is promoted by the United Nations Children’s Fund (UNICEF) and the World Health Organization (WHO) as a communication process that engages and motivates a wide range of partners at all levels to raise awareness of, and demand for, a particular objective [7, 8]. Some specific considerations are required for HPV vaccination [7]. First, while routine vaccination is generally provided to children aged less than 1 year, HPV vaccine is typically delivered to adolescent girls aged between 9 and 13 years, or older if catch-up campaigns are conducted [4]. Providing vaccination to this age group raises issues around informed consent, including how parental consent should be sought prior to vaccination [9–11]. Second, HPV vaccination is primarily delivered to girls rather than both sexes. Third, delivering vaccination at school, a common strategy for HPV vaccination, is novel in some countries [6, 12, 13].

Social mobilisation is intended to increase HPV vaccination acceptability among girls, parents, and respected influencers. Acceptability, the tacit recognition that HPV vaccination is worthwhile, is crucial for high vaccination coverage and effectiveness [14, 15]. Research indicates that mobilisation activities that focus on creating awareness, providing accurate information, building acceptability and sustaining demand for HPV vaccination [9, 10, 16, 17], are most effective in countering the rumours and misinformation that have negatively influenced some HPV vaccination programmes [18, 19].

As part of broader research on lessons learnt from HPV vaccination demonstration projects and national programmes in LMICs [20], this study synthesised social mobilisation and consent experiences and their associations with acceptability.

## Methods

A qualitative study design was chosen, including a systematic review of published and unpublished literature and in-depth key informant interviews [20]. A mapping exercise identified 37 LMICs with at least 1 year of experience implementing HPV vaccine demonstration projects or national programmes as of January 2015. Authors searched five databases systematically for published literature (i.e., Medline, Embase, Global Health, Africa-wide Info, ADOLEC), using a combination of keyword and free-text terms related to HPV vaccination and country names. In total, 1301 abstracts were identified for screening. Authors purposively searched two databases (i.e., ProQuest, Open Grey), the WHO website, and Ministry of Health (MoH) websites of the selected countries for unpublished literature. Authors additionally requested unpublished reports of country experience from country key informants.

Interviews, using a standardized guide, were conducted with in-country representatives to fill country-specific data gaps. After comprehensive review of the literature; 33 countries were approached, 10 country representatives were unresponsive or refused interviews. In total 27 interviews were conducted with representatives from 23 countries. All of the 27 interviewees had a coordinating role in the projects or programmes about which they were interviewed; 16 were Ministry of Health representatives (EPI team members or equivalent), nine were representatives from partner NGOs, and two were doctors/researchers. Interviews were conducted by telephone or in-person, recorded, and transcribed in English as necessary. A topic guide was adapted from WHO’s new vaccine introduction guidelines, it included questions on social mobilisation timing, frequency, messages, materials, target audience; consent processes and reasons for vaccine acceptability or refusal [21].

Data from literature and interview sources were extracted onto an Excel spread sheet using thematic headings and sub-headings developed from WHO’s new vaccine introduction guidelines (e.g., social mobilisation timing, frequency, messages, materials, target audience; type of consent; vaccine acceptability rates and reasons) [21]. Data were analysed thematically using three a priori themes (i.e., social mobilisation, consent, acceptability), while allowing for emerging themes. First-dose HPV vaccination coverage, obtained from survey or administrative reports, was categorised as ‘high’ for above 90 % coverage, ‘medium’ for 70–90 %, and ‘low’ for below 70 % and cross-tabulated with social mobilisation strategies and consent procedures to infer the ‘success’ of the different methods. Reasons for vaccination acceptance and refusal, reported in post-delivery surveys, were ranked highest to lowest by frequency. The three most common reasons in each survey were extracted, scored as ‘3’ for most common, ‘2’ for second-most common, and ‘1’ for third - most common, and scores added to indicate the most common reasons cited across surveys.

## Results

### Extent and nature of sources

In total, 41 published articles, 9 conference abstracts, 124 unpublished documents and 27 key informant interviews (KIIs) were included in analysis, covering 72 HPV vaccine delivery experiences in 37 LMICs. A delivery experience was defined as HPV vaccine delivery to a specific target population (e.g., age range in years, school grade), using a specific vaccination venue (e.g., health facility-based, school-based, outreach, or a combination) within a specific project/programme, as defined by funding source. Not all vaccine delivery experiences collected information covering all data extraction themes.

### Social mobilisation

Data on social mobilisation activities were available for 47/72 (65 %) of HPV delivery experiences in 30 of 37 countries. Overall, almost half (33/72; 46 %) of delivery experiences reported both social mobilisation and first-dose HPV vaccination coverage, of which 19/33 (58 %) reported over 90 % coverage rates (Table 1). Sub-themes related to social mobilisation were target audiences, messages, communication channels, timing and duration, and rumour management.

**Table 1:** Social mobilisation and consent procedures by first-dose HPV vaccination coverage

Social mobilisation and consent	1^st^ dose coverage reported, n (%)^a^		
	>90 %	70–90 %	<70 %
Total number (%) reporting both social mobilisation and first-dose coverage (*n* = 33)^b^	19 (58)	11 (33)	3 (9)
Start of mobilisation prior to vaccination (*n* = 16)^c^			
Within 1–2 weeks of vaccination	2 (50)	2 (50)	0
3 weeks - less than 2 months	3 (60)	2 (40)	0
2–3 months	3 (43)	3 (43)	1 (14)
Message content (*n* = 24)^d^			
Logistics only	4 (67)	2 (33)	0
Informational (logistics and cervical cancer)	9 (82)	1 (9)	1 (9)
Comprehensive (detailed)	4 (57)	2 (29)	1 (14)
Materials and approaches (*n* = 32)^e^			
Interactive	1 (50)	1 (50)	0
Non-interactive	1 (17)	4 (67)	1 (17)
Both interactive and non-interactive	16 (67)	6 (25)	2 (8)
Consent procedures (*n* = 32)^f^			
Written consent by parents/guardians (opt-in)	9 (50)	6 (33)	3 (17)
Implied (opt-out) consent by parents/guardians (opt-out)	8 (73)	3 (27)	0
Changed from written to implied consent	3 (100)	0	0

NB: ^a^Number (%) of delivery experiences that reported first dose HPV coverage; ^b^33/72 experiences reported both first-dose coverage and social mobilisation; ^c^16/33 of these experiences also reported when mobilisation started prior to vaccination; ^d^32/33 of these experiences also reported message delivery methods; ^e^32/33 of these experiences also reported materials; ^f^32/33 of these experiences also reported consent procedures

#### Target audiences

In total, 38/47 (81 %) experiences reporting social mobilisation mentioned target audiences. Primary target audiences were usually parents or girls. Secondary target audiences included communities and credible influencers. Credible community influencers were identified in nine experiences as the most common information sources for parents and included health-workers, teachers/school directors, community or religious leaders and influential family members [6, 22]. Credible national influencers included members of royalty, wives of elected officials, national and sub-national political leaders, and entertainers (e.g., television stars), who were encouraged to launch campaigns and champion HPV vaccination to increase confidence and interest among both primary and secondary target audiences [23–25]. When the MoH was involved, social mobilisation typically started at national level, gaining support of national influencers [26].

#### Messages

Approximately half (24/47; 51 %) of experiences reporting social mobilisation also reported message content (Table 1). The vast majority of messages were framed around the ‘anti-cancer’ benefits of vaccination rather than prevention of a sexually transmitted infection (STI). Messaging was categorised as (i) logistical only, e.g., vaccination venues, dates, eligibility; (ii) informational, e.g., logistical information plus basic descriptions of vaccine safety and cervical cancer risk; or (iii) comprehensive, e.g., providing further detailed information on cervical cancer epidemiology and/or the extent of vaccine action against HPV. Informational messages, which combined vaccination logistics and simple explanations of cervical cancer and vaccine safety, were associated with highest coverage compared with either simply logistical or fully comprehensive messages (Table 1). For example, the information required was described by one KII:

*“Parents thought the vaccine was new, has not been widely used and may have some health consequences. They had also other questions that required explaining,* e.g., *why children of a certain age and only girls are being vaccinated. Could the vaccine affect their fertility?” (KII, Country 26)*

#### Communication approaches

In total, 33/47 (70 %) experiences reporting social mobilisation also reported on communication approaches used (Table 1). Various approaches and materials were used to reach target audiences. Communication approaches were categorised as interactive (e.g., one-to-one or group meetings at schools or health facilities, home visits by health-workers) or non-interactive (e.g., leaflets, posters, loud-speaker, radio, or television announcements). Over half of experiences using interactive approaches (17/26) achieved high first-dose coverage (over 90 %) compared to (1/6) 17 % for those using only non-interactive approaches (Table 1). For example, parents in one experience who reported they had attended a teacher-parent meeting about vaccination were more likely to have a vaccinated daughter compared to parents who did not report attending meetings [22]. While interactive approaches were reportedly more successful than non-interactive in influencing HPV vaccination uptake, most experiences (24/33; 73 %) used a combination of communication approaches [26]. For example:

*“We held two meetings at school, but parental involvement was a bit difficult; not many came because they were busy with their jobs. And then through TV or radio, they were informed” (KII, Country 5)*

*“Vaccination days were announced by radio but everyone has a mobile phone and we could have made better use of texting.” (KII, Country 25)*

#### Timing and duration of activities

In total, 16/47 (34 %) experiences reporting social mobilisation also reported on timing of mobilisation activities. Seven experiences started 2 to 3 months prior to commencing vaccination, while nine others started less than 2 months before vaccination. Timing did not appear to be correlated with vaccine coverage achieved (Table 1); however, KIIs reported they had encountered problems during delivery when mobilisation had been conducted less than a month before vaccination:

*“Social mobilisation needed more time (than 2 weeks before vaccination day) - it should start earlier and continue until the last vaccination day.” (KII, Country 3)*

Only 4/47 (1 %) experiences described duration and/or frequency of mobilisation activities. Activities, including community drama and/or radio broadcasts, were provided for 1 day or continuously over 1 to 2 weeks at vaccination venues. Social mobilisation activities sometimes intensified as vaccination day approached:

*“Mobilisation varied depending on the method used to deliver the message or timing prior to actual vaccination… mobilisation was conducted in the same community once a week before vaccination started, to daily on the vaccination week.” (KII, Country 33)*

#### Rumour management

Rumours, reported in 13/37 (35 %) countries, were generally consistent in content and often spanned more than one delivery experience per country. One country reported rumours spreading from a neighbouring country. Rumours generally focused on whether HPV vaccine affected fertility, caused dangerous side effects, or was experimental (Table 2) [22, 27].

**Table 2:** Reported rumours, institutional refusals, and management approaches

Reported rumours	Management approaches (preventative and reactionary)
HPV vaccine is experimental/untested (Countries 3, 12, 24)	• Rumours resulted from opt-in consent, which was changed to opt-out; • Government and experts immediately addressed rumours.
HPV vaccination causes fertility problems (Countries 8, 17, 21, 24, 31, 16, 28)	• Mobilisation was started very early and messages built into parent-teacher meetings; • High-level advocacy using parliamentarians from the beginning of the programme; • Intense mobilisation targeted anti-vaccination lobbyists; • A reactive crisis response was organised, including meeting with communities.
Vaccine causes long-term adverse events, e.g., death, cancer (Countries 28, 33, 35, 26)	• Adverse events were investigated and guardians reassured that it was not due to vaccination.
There is another cure for cervical cancer other than vaccination (Country 35)	• Rumours were tackled immediately with email newsletter and/or parent meetings.
Institutional refusals related to the vaccine	Management approaches
Private/faith-based schools (Countries 23, 24, 31, 35, 37)	• Sensitization through the community and targeted mobilisation using influencers; • Media access to correct information so communities could obtain HPV vaccine information from an independent source.
Churches/religious groups (Countries 3, 28, 37)	• Increased face-to-face, community, and religious leaders’ meetings.
Community/parent groups (Countries 1, 5, 6, 10, 14, 18, 23)	• Identified groups opposing vaccination were provided with more information; • Frequent repetition of messages; • Involved leaders and managed vaccination through government system; • Provided additional training and information to health-workers and teachers.
Anti-vaccination lobbyists, human rights groups, academics (Countries 12, 30)	• Provided additional media information and internet-based information campaigns.
Teacher and health-worker reluctance to vaccinate girls (Countries 6, 23)	• Provided additional training to healthworkers and used peers to trace missing and out-of-school girls.

Experiences generally attempted to prevent rumours and institutional refusals (e.g., refusal by schools or religious groups) by conducting intensive and repeated sensitization activities (Table 2). Once rumours arose, credible influencers were often mobilised to counteract misinformation with targeted messages in specific communities (Table 2). Government endorsement was also reported as useful in mitigating rumours and increasing vaccine acceptability if uptake was low [6, 22, 26, 28], e.g., some experiences distributed government or WHO letters of vaccine endorsement to allay parental concerns (Table 2). However, in two experiences, rumours and misinformation caused the withdrawal of government endorsement and HPV vaccination was suspended [18]. Delays in addressing rumours, especially those around severe side effects or that the demonstration project was actually a clinical trial, resulted in vaccine delivery being delayed in one country and stopping prematurely in two countries, including India [18]. A prompt response was critical for success, for example:

*“[Rumours were] usually due to confusion around girls being sterilised and other misconceptions. [They said] ‘Once again women are being used to pilot new products’. [Rumours were] overcome through intensive health messages and education.” (KII, Country 2)*

### Consent

In total, 50/72 (69 %) delivery experiences reported consent procedures, 32 (64 %) of which also reported first-dose HPV coverage. Of these, 18/32 (56 %) used opt-in written parental consent, 11/32 (34 %) used opt-out implied consent, and three (9 %) changed from written to implied consent during implementation. Opt-in written consent was defined as parents or guardians actively giving permission for daughters to be vaccinated by returning a signed consent form. In addition to signing a consent form, some experiences required parents/guardians to accompany daughters to vaccination venues. In three countries, girls were not vaccinated if the parent/caregiver was not present [29]. Two national programmes had added HPV vaccine to a pre-existing school health programme consent sheet that included a range of health interventions delivered to children over 5 years old. Opt-out implied consent meant all girls were vaccinated except those whose parents or guardians formally refused vaccination or kept girls home on vaccination days [10].

*“[Written consent] is not used routinely, we developed it for the new vaccine. The MOH advised us to use consent as it was the first time the vaccine was used in the country” (KII, Country 6)*

Parents questioned why opt-in consent was required for HPV vaccine but not others, such as measles or hepatitis vaccines, and 13 experiences reported that using opt-in consent resulted in rumours that the HPV vaccine was experimental. Ten of these 13 experiences had data on uptake rates; four reported 64–70 % uptake and six reported 70–90 % uptake. In addition to concerns about vaccine safety, one informant described parental concerns that signing a written consent form indicated they took full responsibility for any consequences, including adverse events:

“*For the girls to be vaccinated the project required a lengthy consent form to be signed and returned to the school. Parents/guardians felt that by signing the form they would shoulder the blame in case of daughter’s death or emergency following vaccination” (KII, Country 14)*

*“Consent was opt-in for first year, which caused a lot of challenges and suspicion” (KII, Country 2)*

Delivery experiences using opt-out consent were more likely to report higher coverage than those using opt-in consent (Table 1). Lengthy consent procedures were reported to reduce vaccination uptake, and eleven countries proposed simplifying consent forms or changing to implied consent [25, 27]. One reported uptake increased from 77 to 99 % on switching from opt-in to opt-out consent, although other programme factors also changed in the same time period. Another, on comparing uptake using opt-in and opt-out strategies, switched to opt-out as it drastically increased uptake. Four countries changed to opt-out consent because of rumours about the vaccine being experimental and to better align with national consent policy for routine immunisation [25].

It should be noted that girls were not always passive in the vaccination decision. Four countries reported that girls who wanted to be vaccinated had persuaded their parents/guardians to sign consent forms [11]. One experience allowed children aged 12 years and above to legally consent to vaccination [30]. Four countries reported that, despite parental consent, some girls refused to be vaccinated.

### Acceptability

In total, 45/72 (62 %) experiences reported on HPV vaccine acceptability in 23 of 37 countries. Of these, 14/45 (31 %) experiences in nine countries formally measured acceptability in surveys, studies or post-introduction evaluations; the remainder reported acceptability without detailed methods. Studies in three countries measured vaccine acceptability as parents’ and/or girls’ willingness to be vaccinated and subsequent uptake of HPV vaccination [31–33]. One study in one country specifically selected vaccinated and unvaccinated girls to survey [22]. Acceptability in the remaining five countries was measured in community coverage surveys [6, 26, 27, 30, 34–37]. One study also measured acceptability among health workers as nurse’s willingness to recommend HPV vaccination [38]. Publications on experiences in Brazil, Cameroon, Kenya, Peru, South Africa, Tanzania, Uganda, and Vietnam reported parental acceptability [6, 22, 27, 30–32, 37–39]. Acceptability among girls was reported in Cameroon, Tanzania, Uganda, and Vietnam [22, 26, 33, 35, 36]:

*“Interestingly, girls who did not normally attend schools would often come to the school on vaccination day to receive their vaccines.” (KII, Country 25)*

Studies of hypothetical acceptance prior to vaccination were excluded from this study; the reason was illustrated by a Kenyan study that reported low first-dose uptake despite recording high parental willingness to vaccinate at baseline [32]. The most common reasons reported in post-vaccination surveys for parental acceptance or refusal of HPV vaccine are shown in Table 3. Communicating directly with influencers such as family members, teachers and health-workers was reported as strongly associated with acceptability [22, 26, 27, 31], as was being well-informed about HPV, HPV vaccination, and cervical cancer [27, 30, 32, 35, 40]. Exposure to rumours and incorrect or limited information about HPV vaccine safety, side effects and association with cervical cancer, were associated with low acceptability [27, 33, 35, 37–39].

**Table 3:** Reasons for accepting or rejecting HPV vaccination reported in surveys in 8 countries^a^

Reasons for acceptance stated by parents/guardians	Score^b^	Surveys (n)
Protection from cancer	23	8
Vaccination is good for health	22	8
Perceived cervical cancer risk or susceptibility	8	3
Convincing information	6	3
Vaccine is safe	5	2
Following others’ advice	5	3
Protection from infection	5	4
Informed about the programme	4	2
Vaccine is free	3	2
To avoid shame/stigma of an STI infection	2	2
Interest in HPV vaccine and education	2	1
Heard of cancer/knowledge of someone with cancer	1	1
Perceived severity of infection and consequences	1	1
Provided at school to every child	1	1
Reasons for not starting stated by parents/guardians		
Lack of motivation		
Fear of adverse effects on fertility and vaccine safety	16	8
Girls or parents do not want vaccine	6	3
May encourage early sex	4	2
Cancer considered low severity/low risk	3	1
Concern about vaccine effectiveness	3	1
Undisclosed reasons	2	1
Perceived low risk of infection	2	1
Not good for a child	1	1
Lack of information		
Not aware of the programme	13	6
Insufficient information	8	4
Systems barriers		
Absenteeism	15	7
Difficult to determine age eligibility	9	7
Location and time not convenient	2	1
Health provider didn’t recommend	1	1

^a^Fregnani JHTR, 2013; Kury CMH, 2013; Wamai RG, 2012; Wamai RG, 2012; Vermandere H, 2014; Botha MH, 2014; Watson Jones D, 2012; Katagwa, 2014; Galagan, 2013

^b^The most common reason cited within each survey was given a score of 3, the second most common scored 2 and the third most common reason scored 1. Reasons were then pooled with their scores to indicate the most commonly cited reasons across all surveys

Vaccination refusal by private schools, religious groups and health professionals was reported in 15 countries (Table 2). In five experiences, some school principals or teachers would not allow vaccinators into their schools due to fear of parent reactions, religious beliefs, or rumours [22, 41]. In two experiences, the school administration thought that they would be held responsible for any severe adverse effects. In Tanzania, parental refusal was higher in private than public schools, as school management feared loss of revenue if parents objected to vaccination [42]. In one experience, school administrators in 23 schools refused access to health-workers as some parents did not consent to vaccination. The importance of training key influencers in the community was emphasized by many KIIs:

*“At first religious leaders were not specifically targeted for mobilisation but we soon realised they had a powerful influence in the communities so they were included in the social mobilisation during the first year. Low level of social mobilisation of out of school girls yielded low uptake at community outreach events and at the health facility. Rumours may have also had an influence but after this was realised social mobilisation was increased.” (KII, Country 22)*

*“The anxiety among the health workers in the first campaign was high as they thought it was potentially dangerous. [We] need to spend a lot more time with health professionals to educate them fully” (KII, Country 30)*

Systems barriers, not necessarily related to acceptability, also affected uptake. For example, school absenteeism on vaccination days was reported as a major barrier to HPV vaccine uptake [6, 28, 32, 35, 37, 42, 43]. Additionally, inadequate official communication about vaccination timing and/or venue and difficulties determining age eligibility were reported by experiences in India, Kenya, Nepal, Peru, Uganda, and Tanzania [6, 28, 32, 42–44].

## Discussion

### Key findings

Social mobilisation data were available from 65 % of delivery experiences covering 30 LMICs. Sources reported social mobilisation was essential for high HPV vaccine acceptability and coverage, though few experiences formally evaluated different mobilisation strategies [6]. Approaching a range of target audiences, including girls, parents, and influencers, appeared to help broaden support for HPV vaccination. Focusing messages on cervical cancer protection, rather than STI prevention, appeared to help minimise sensitivities around girls’ sexuality [9, 10, 16, 17]. Grey literature identified interactive communication as the most effective when parents were asked about their sources of information about the vaccine and influencers on their decision. While few experiences reported on timing and duration of mobilisation activities, early initiation and increasing intensity appeared most effective. Social mobilisation for HPV vaccination, especially during introductory phases and alongside provider training on risk communication, was reported as more rigorous and frequent than mobilisation for routine infant vaccination to mitigate risks associated with rumours [3, 7, 21, 45]. Rumour management was a key aspect of social mobilisation, requiring both preventive sensitisation and rapid reactive risk mitigation [18, 19].

Consent data were available from 69 % of experiences. Written opt-in consent was common for HPV vaccination, due to WHO recommendations on consent [7]. However, opt-in approaches sometimes contributed to parental worries about vaccine safety, particularly in countries using opt-out, implied consent for routine vaccination. Forms could be lost or submitted late, even among households that did not object to HPV vaccination. Implied opt-out consent was associated with higher first-dose coverage, as it was more often in-line with other routine immunisation procedures and national policy, though causation cannot be assumed.

Acceptability data were available from 62 % of experiences. HPV vaccination was generally well accepted among individuals and communities in LMICs. Similar acceptability rates have been reported for other newly introduced vaccines targeting adolescents and school-age children in high-income countries, such as Tdap, meningococcal conjugate (MCV4), and influenza vaccines [46, 47]. The role of national and community influencers supports findings for other vaccines targeting adolescents, including Tdap and MCV4 [48].

### Implications

The positive association between intensive, interactive social mobilisation and first-dose coverage supports existing communication recommendations around vaccine introduction strategies [7, 21]. Formative research prior to the development of communications strategies provided generally consistent results across HPV delivery experiences, indicating further formative research on effective communication strategies in other LMICs might not be needed [49–51]. Findings indicated that social mobilisation and training of ‘credible influencers’ should start sufficiently early to allow parents/guardians time to consult well-informed sources [7, 17, 27]. While mobilisation timing was not associated with first-dose uptake, many factors influence uptake and the importance of timely social mobilisation was a common theme in interviews. WHO recommends initiating social mobilisation 3 months prior to starting HPV vaccination [7]. Findings indicated that particular mobilisation efforts should focus on out-of-school girls and non-government (private) or faith-based schools, where refusals were highest, particularly in countries where primary delivery strategies are school-based.

Informed consent is enshrined in medical ethics and international human rights law [52]. While those below the age of consent should be protected and informed consent ethics respected [10, 53, 54], the requirement for opt-in written parental consent for HPV vaccination has been reported as problematic. The requirement for parental consent can affect utilisation of adolescent health services and the potential negative impacts on vaccination uptake must be considered [9, 10, 55]. Where opt-in written parental consent is used, particularly if this differs from routine vaccination, reasons should be explained clearly and procedures kept straightforward [10, 56]. Some countries have already reduced the age of consent to enable girls to choose HPV vaccination [10, 30]. Opt-out, implied, consent was acceptable in countries in which it was documented and associated with fewer difficulties, and thus several countries have changed from opt-in to opt-out consent. Informed consent procedures for routine healthcare interventions in many LMICs need to be improved [10], developing a school health programme consent sheet that is signed at school enrolment may be the most efficient option for school-based HPV vaccination.

Acceptability was most readily affected by rumours and misinformation. Rumours were remarkably similar in content across geographical regions. This suggests messages could be tailored from the outset to address concerns about experimental vaccines and effects on fertility, to pre-empt anticipated concerns [44, 57, 58]. Experiences from Uganda and Peru indicated that simple messages, emphasizing health benefits to girls, were as effective in gaining high vaccination uptake as more complicated messages [59]. Similarly, simplified messages were found to increase uptake of measles, mumps, and rubella vaccination for infants [60], supporting the communications benefits of simple, clear messaging.

### Limitations

This study had several limitations. First, approximately 30 % of experiences did not report on social mobilisation, so it was unclear whether social mobilisation had been done or how effectively. Second, depth and quality of social mobilisation data, especially in unpublished reports, varied considerably. Interviews helped to fill gaps in these reports, but were usually conducted with EPI managers or project/programme representatives who were not always familiar with all aspects of social mobilisation implementation, consent, and acceptability. Third, associations found between mobilisation and first-dose coverage cannot be interpreted as causal, as sources used different study designs and outcome measures. Countries with poorer social mobilisation (e.g., non-interactive approaches) may have had weaker health systems, less money to deliver sophisticated mobilisation, and/or ineffective vaccine delivery strategies. Implementation and reporting of delivery experiences was heterogeneous, with many factors influencing first-dose coverage.

## Conclusions

HPV vaccine delivery experiences in LMICs to-date underscore the value of effective social mobilisation. To allow sufficient time to address concerns and misconceptions, mobilisation should be initiated as soon as possible and conducted systematically. Mobilising credible influencers, who are trusted by the community, as well as national figureheads and/or celebrities, can increase public confidence and therefore vaccination coverage. Simple messages that build interactively on existing knowledge of cancer risks appear more effective than either complex or non-interactive approaches. Consent procedures should be considered carefully, to balance informed consent with public health benefits. High acceptability and uptake of HPV vaccination was achievable despite minimal vaccination and health knowledge in many communities.

## Abbreviations

HPV: Human papillomavirus
KII: Key informant interview
LMIC: Low and middle-income country
MoH: Ministry of Health
WHO: World Health Organization
